# Superior long‐term stability and function associated with femoral cortical button versus interference screw fixation in ACL reconstruction: A systematic review and meta‐analysis

**DOI:** 10.1002/jeo2.70439

**Published:** 2025-09-24

**Authors:** Jonathan Elias, Mitchell Kaplan, Michael Bickford, Thomas Oliver, Kunal Shah, Elizabeth Ford, Sean McMillan

**Affiliations:** ^1^ Rowan‐Virtua School of Osteopathic Medicine Stratford New Jersey USA; ^2^ Inspira Health Network Vineland New Jersey USA; ^3^ Virtua Health System Marlton New Jersey USA

**Keywords:** ACL repair, cortical button, femoral fixation, hamstring tendon graft, interference screw

## Abstract

**Purpose:**

There are two main methods of femoral graft fixation during anterior cruciate ligament reconstruction (ACLR): cortical button (CB) and interference screws (IS). Each fixation yields its own unique outcomes; however, there is yet to be an established gold standard. We seek to compare femoral CB to IS fixation in ACLR using all soft‐tissue, autograft hamstring grafts. We hypothesise that there will be no significant differences between the two methods.

**Methods:**

A systematic review and meta‐analysis were conducted following the 2020 PRISMA guidelines. PubMed, Cochrane, Embase, Web of Science and Scopus were screened for potential randomised studies. The studies must have utilised both the semitendinosus and gracilis, and utilised either metallic or bioabsorbable IS, or fixed or adjustable loop CB fixation on femoral fixation. All studies in both groups must have used interference screws on the tibial side. The 2‐year follow‐up analysed KT‐1000 side‐to‐side differences, while the 5–10 year follow‐up compared Lysholm and Tegner scores.

**Results:**

A total of 12 randomised studies were included in the final systematic review, providing an evaluation of 583 distinct patients. Two of the studies were excluded from the meta‐analysis, due to not providing pre‐ and post‐operative means and standard deviations. At 2‐year and 5‐10 year follow‐ups, the mean ages were 30.5 ± 8.7 and 30.2 ± 8.1, respectively. Analysis of the 2‐year follow‐up favour of CB (*p* = 0.01) in regards to the reduction in KT‐1000 side‐to‐side differences. Analysis of the 5–10 year follow‐up revealed favour of CB in regards to the Lysholm Score (*p* < 0.01), and the Tegner Score (*p* < 0.01).

**Conclusions:**

At 2‐years postoperatively, femoral fixation utilising cortical buttons portrayed less knee laxity than interference screws. At the 5–10 year follow‐up, cortical buttons yielded more sports and work‐related activity, less pain, and overall greater function than did the interference screws.

**Level of Evidence:**

Level II.

**Abbreviations**
ACLR: anterior cruciate ligament reconstruction
CB: cortical button
HT: hamstring tendon
IKDC: International Knee Committee Documentation
IS: interference screw

## INTRODUCTION

Anterior cruciate ligament reconstruction (ACLR) is predominantly performed to address knee instability after complete rupture of the anterior cruciate ligament (ACL) [3]. This procedure aims to restore structural integrity and knee kinematics through the use of a graft in place of the disrupted native ACL [1]. Although bone‐patellar tendon‐bone (BPTB) is considered the ‘gold’ standard of care, hamstring tendon (HT) and quadriceps tendon (QT) autograft‐based reconstruction have evidence supporting their status as valid alternatives [6, 11].

Regardless of the graft utilised, there are two commonly utilised methods of fixation for the graft to be secured within the femoral tunnel. These fixation options are interference screws (IS) and cortical button (CB) suspensory fixation. Interference screws, commonly composed of either metal, Polyether Ether Ketone (PEEK), or bioabsorbable materials, function to provide compressive aperture fixation within the femoral bone tunnel [5, 26]. These screws serve to compress and secure the graft against the bone tunnel wall, allowing for time zero fixation [22]. Benefits of IS fixation during ACLR include reduced tunnel widening, graft‐tunnel motion, and graft creep [17]. In comparison, CB devices are comprised of a metallic button and a looped construct that allows for the graft to be fixated at the far cortex of the femur through a suspensory mechanism [16]. A benefit to this form of fixation is circumferential contact of the graft can be obtained by the graft within the femoral tunnel for increased biologic healing potential [20].

In this systematic review and meta‐analysis, we compare the outcomes of femoral fixation between IS to CB suspensory devices in ACLR patients receiving HT autografts. It is hypothesised that no significant differences between IS and CB fixation for patients undergoing ACLR with hamstring autografts would be observed.

## METHODS

A systematic review and meta‐analysis were conducted following the 2020 Preferred Reporting Items for Systematic Reviews and Meta‐Analyses (PRISMA) guidelines [21]. We sought to compare the use of IS to CB fixation on the femoral aspect during ACLR, using all soft‐tissue, autograft hamstring tendon grafts. To investigate postoperative outcomes, we compared KT‐1000 side‐to‐side differences, Tegner scores, and Lysholm scores between the two proximal fixation groups. KT‐1000 side‐to‐side differences were assessed at a 2‐year follow up, while Tegner and Lysholm scores were assessed at 5–10 years postoperatively.

### Search procedure

On 15 August 2024, five online databases (Cochrane, Embase, PubMed, Scopus and Web of Science) were searched using the following search string: (‘Cortical Fixation’ OR ‘Suspensory Fixation’ OR ‘Fixed loop’ OR ‘Interference screws’ OR ‘Arthroscopic technique’ OR ‘Screw fixation’) AND (‘ACL Reconstruction’ OR ‘Anterior Cruciate Ligament Reconstruction’ OR ‘ACLR’ OR ‘Arthroscopic anterior cruciate ligament reconstruction’ or ‘Bone‐tendon‐bone’ OR ‘Bone‐hamstring‐bone’) AND (‘Semitendinosus’ OR ‘Hamstring’) AND (‘Randomised Controlled Trial’ OR ‘RCT’ OR ‘Clinical Trial’). Key terms were identified using the Medical Subject Headings (MeSH) tool.

### Inclusion and exclusion criteria

Included studies must have met all of the following criteria: randomised, conducted ACLR using a quadrupled single‐bundle hamstring tendon autograft composed of both the semitendinosus and gracilis tendons, utilised either metallic or bioabsorbable interference screws (IS group) or fixed or adjustable loop cortical button (CB group) fixation on the femoral aspect of the ACLR procedure, used interference screws on the tibial aspect in order to control for confounding variables, published after 2010. Studies were excluded if allograft tendon grafts or the all‐inside ACLR approach were used. All cross‐sectional, cohort, case control, case series, case studies, cadaveric, and nonrandomized studies were excluded.

### Study selection

The search string yielded 309 total articles across the five databases. The results retrieved from each database using the search string were imported into Rayyan.ai. Automated detection of duplicates was utilised to assess for any potential duplicates; however, the detected duplicates were reviewed by two independent authors to confirm its findings. Duplicates were then excluded, and the remaining studies were subjected to evaluation by two independent authors for determination of inclusion and exclusion. Any dissent was resolved by a third author.

Following deletion of duplicates, 163 articles remained. The abstracts and titles of the remaining studies were screened and 126 studies were eliminated in the process. Thirty‐seven records were then sought for retrieval and successfully retrieved. Following retrieval, the 37 studies underwent a thorough, full‐text examination. Twenty‐five studies were eliminated during full‐text review, due to 13 having insufficient data, three not including both semitendinosus and gracilis tendon grafts, three using double‐bundle ACLR, two not using interference screws on the tibial attachment, two using the all‐inside approach, and two having a follow‐up time outside of our study.

### Data extraction

For the included studies, all necessary data was extracted onto a Microsoft Excel sheet. All data was collected independently by four reviewers. The titles, authors, year of publication, country, study design, recruitment period, interventions, sample size of each intervention, ages, inclusion criteria, outcomes collected and main findings were collected from each study. Additionally, the following variables were extracted in mean and standard deviation (SD) format: 2‐year KT‐1000 scores, 5–10 year Tegner scores, 5–10 year Lysholm scores. If a study included additional groups that were not of interest to our review, only groups that met our inclusion criteria were included in this review and analysis.

### Statistical analysis

All statistical analysis was conducted on IBM Statistical Package for Social Sciences (SPSS) version 29. A measure of effect size regarding KT‐1000 side‐to‐side differences, Tegner scores, and Lysholm score was analysed utilising a random effects model. A subgroup analysis was conducted in order to assess for significant differences between the two groups. A Δ Hedge's *g* value ≥ 0.80 was used as a cutoff to assess for a clinically significant difference, and a *p*‐value < 0.05 was used to assess for statistical significance, using 95% confidence intervals.

Heterogeneity was analysed using the *I* ^2^, *H* ^2^, and *τ* ^2^ values from the measure of effect size using the KT‐1000 side‐to‐side differences, Tegner score, and Lysholm scores using a random effects model.

### Risk of bias and certainty of evidence assessment

Bias in the articles was assessed independently by two authors. Because all of the articles included were RCTs, they were subjected to evaluation according to the Risk of Bias‐2 (RoB‐2) tool. To assess for quality, all included studies have been also subjected to a modified version of the Grading of Recommendations Assessment, Development and Evaluation (GRADE).

## RESULTS

### Search results

**Figure 1:**
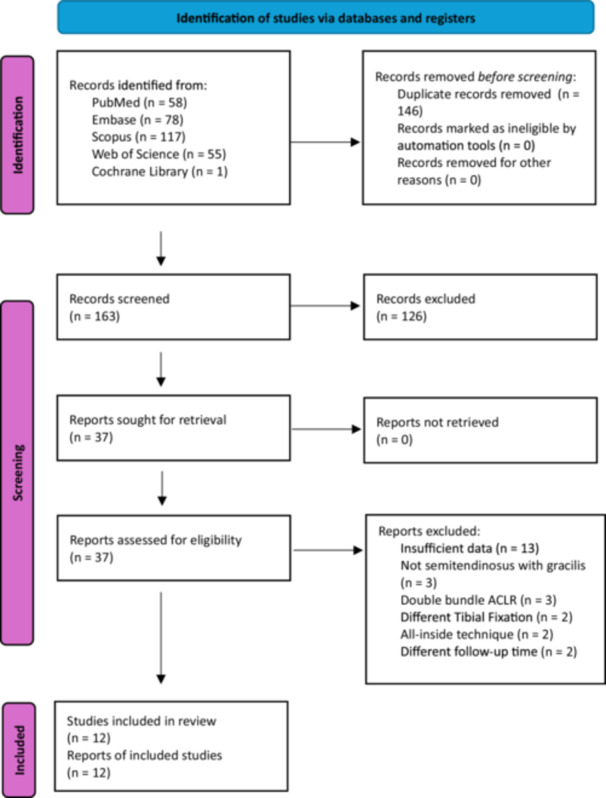
PRISMA flowchart of article selection.

A total of 12 randomised controlled trials (RCT) were included in the final systematic review [2, 4, 7, 10, 13, 14, 19, 23, 27, 29, 30, 31]. A PRISMA flow chart of the selection process is portrayed in Figure 1. Two studies conducted by Stener et al. [27] and Djordjević et al. [7] were included in the systematic review, but were excluded from the meta‐analysis, due to not providing pre‐ and post‐operative means and standard deviations regarding their data. An RCT conducted by Roger et al. was not included in either the systematic review or the meta‐analysis, due to using femoral pin transfixation, rather than a cortical button [24].

### Group demographics

The 10 studies included in the meta‐analysis yielded an evaluation from follow‐ups from 537 distinct patients. At 2‐year and 5–10 year follow‐ups, the mean ages were 30.5 ± 8.7 and 30.2 ± 8.1, respectively. Baseline KT‐1000, Lysholm scores, and Tegner scores are presented in Table 1.

**Table 1:** Baseline mean ± SD regarding KT‐1000 side‐to‐side differences, Lysholm scores, and Tegner scores between the two groups.

Measure	IS	CB
Preoperative		
KT‐1000 side‐to‐side difference (mean ± SD)	4.2 ± 2.7	6.9 ± 2.32
Lysholm (mean ± SD)	64.7 ± 16.7	60.0 ± 12.0
Tegner score (mean ± SD)	4.0 ± 1.1	3.0 ± 1.3

Abbreviation: SD, standard deviation.

### Effects of intervention

**Figure 2:**
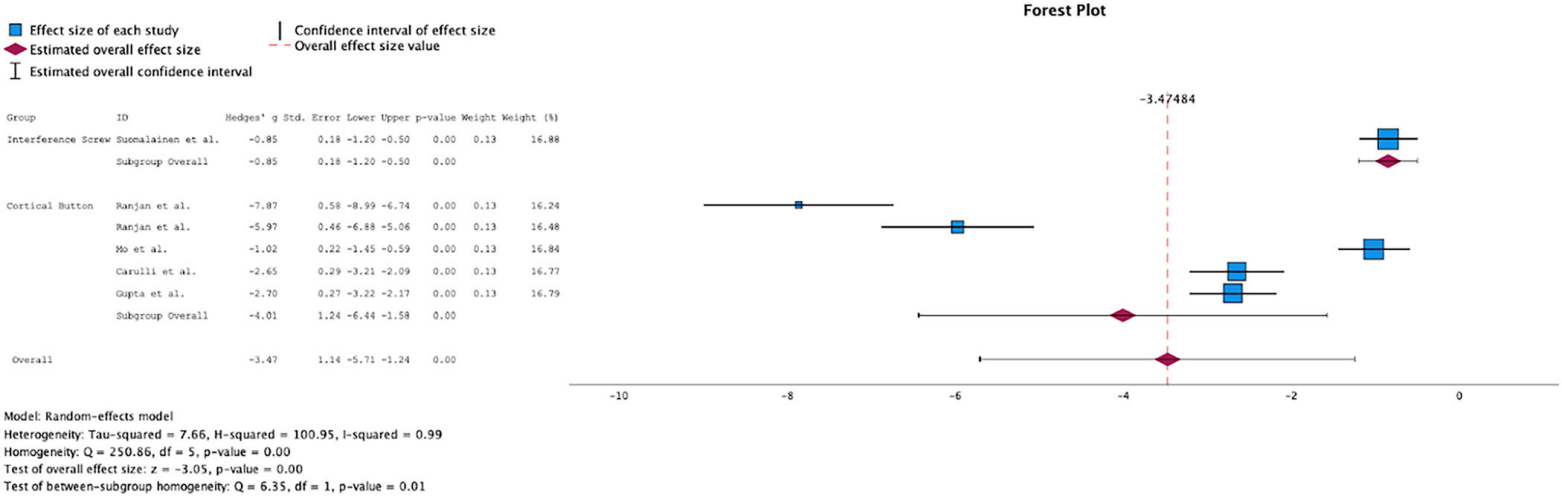
Comparison of KT‐1000 side‐to‐side differences at 2‐year follow‐up.

**Figure 3:**
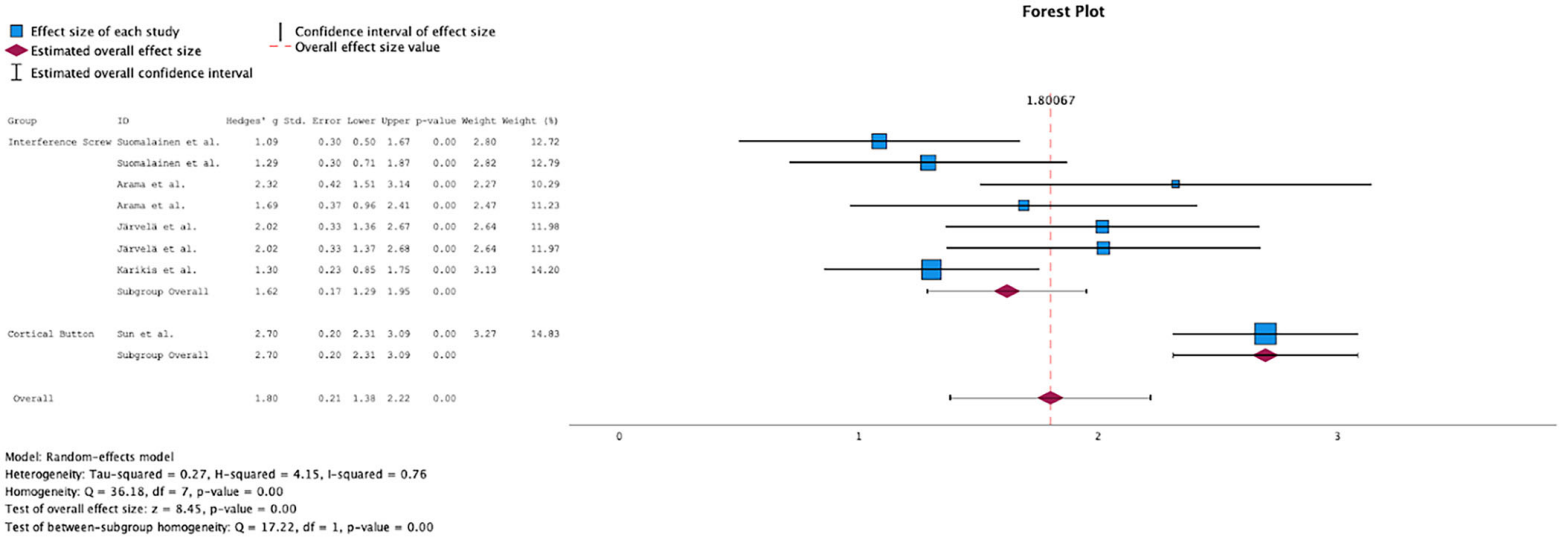
Comparison of Lysholm scores at 5–10 year follow‐up.

**Figure 4:**
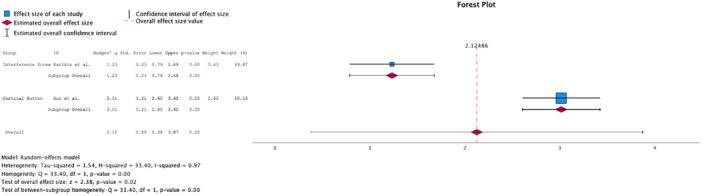
Comparison of Tegner scores at 5–10 year follow up.

Analysis of the 2‐year follow‐up portrayed a significant difference in favour of CB (Δ Hedge's *g* = 3.16, *p* = 0.01, Figure 2) regarding the reduction in KT‐1000 side‐to‐side differences. Analysis of the 5–10 year follow‐up revealed a significant difference in favour of CB regarding the Lysholm Score (Δ Hedge's *g* = 1.08, *p* < 0.01, Figure 3), and the Tegner Score (Δ Hedge's *g* = 1.78, *p* < 0.01, Figure 4). The results have been summarised in Table 2.

**Table 2:** Summary of the analysed data between IS and CB.

Variable	Group	Mean ± SD	Hedge's *g*	Δ Hedge's *g*	*p*‐Value
2‐year follow‐up					
KT‐1000 side‐to‐side difference	IS	2.0 ± 2.4	−0.85	3.16	0.01*
	CB	1.4 ± 1.8	−3.47		
5‐10 year follow‐up					
Lysholm	IS	89.4 ± 15.2	1.62	1.08	<0.01*
	CB	89.0 ± 9.0	2.70		
Tegner	IS	5.6 ± 1.5	1.23	1.78	<0.01*
	CB	7.7 ± 1.8	3.01		

*Note*: The use of *signifies statistical significance. The use of red font signifies clinical significance.

Abbreviations: CB, cortical button; IS, interference screws; SD, standard deviation.

### Risk of bias and certainty of evidence assessment

A Grading of Recommendations Assessment, Development and Evaluation (GRADE) analysis and certainty of evidence of the included 12 studies was performed independently by two authors based on Cochrane guidelines [25]. Out of those 12 studies, three studies were assessed as low quality of evidence, and the remaining nine studies were of moderate quality of evidence. Each study was critically appraised, and criticism notes are provided in Table 3.

**Table 3:** Summary of GRADE analysis of the included studies based on Cochrane guidelines.

Author, year	Study design	Risk of bias	Inconsistency	Indirectness	Imprecision	Publication bias	Final grade	Criticism
Suomalainen et al. [30], 2012	RCT	Serious	Serious	Serious	Very serious	Not likely	Low	While the study was well‐designed for its methodology, there was significant data loss at follow‐up, ranging from 3% to 30% of patients in various groups. As well as there are about 10% of patients who experienced graft failure, resulting in loss of that data, which was not part of the statistics, representing a better design of statistics, but loss of data can impact the power of the data.
Ranjan et al. [23], 2018	RCT	Not serious	Serious	Serious	Not serious	Not likely	Moderate	A well‐designed study reported all the outcomes mentioned in the design, with minimal patient loss to follow‐up. Outcomes, patient loss to follow up were appropriately accounted for, and statistics were reported for all the outcomes measured in a respective fashion.
Mo et al. [19], 2024	RCT	Not serious	Serious	Serious	Not serious	Not likely	Moderate	Overall study provides excellent data. A well‐organised methodology, but concerns arise when a patient with a concomitant procedure was decided to be included for meniscus tears, could be a confounding variable in reported outcomes. All patients were accounted for, and outcomes were reported appropriately.
Suomalainen et al. [31], 2011	RCT	Not serious	Serious	Serious	Serious	Likely	Low	The study provides great insights in well‐organised outcomes as mentioned in the methods. The only concern that arises is that the included sample size was less than that of the power initially calculated by the statistician, and about 20% of the patients were lost to follow‐up.
Stener et al. [27], 2010	RCT	Not serious	Serious	Serious	Not serious	Not likely	Moderate	Well‐designed study with no deviation from any measurement or intended outcomes. The only drawback power analysis was not performed for the measurement required sample size for the study.
Arama et al. [2], 2015	RCT	Not serious	Serious	Serious	Not serious	Not likely	Moderate	Structured clinical study with appropriate randomisation and clinical assessments performed and reported.
Järvelä et al. [13], 2017	RCT	Not serious	Serious	Serious	Not serious	Not likely	Moderate	Well‐organised study with randomisation, measurement, and reporting of pre‐ & post‐intervention data. Power analysis for sampling was not reported in the methods, but all patients lost at follow‐up were accounted for.
Carulli et al. [4], 2017	RCT	Not serious	Serious	Serious	Not serious	Likely	Low	The study provides insights into the use of fixation techniques, but the problem there stems from the randomisation process not being clearly described, and outcomes are not completely reported at 6 months and 1 year, as mentioned in the methods.
Karikis et al. [14], 2017	RCT	Not serious	Serious	Serious	Serious	Not likely	Moderate	The study design lacks clarity in the randomisation process as well, and no data is reported for 2‐year outcomes. There is a loss of patient follow at 10‐15% in subgroups. Concerns arise with the possibility of a lack of power for the study.
Sun et al. [29], 2011	RCT	Not serios	Serious	Serious	Not serious	Not likely	Moderate	The study is well‐designed with randomisation and reporting of outcomes at the mentioned intervals.
Djordjević et al. [7], 2021	RCT	Not serious	Serious	Serious	Not serious	Not likely	Moderate	The study was well designed, with proper randomisation, and all the outcomes mentioned were measured and reported.
Gupta et al. [10], 2017	RCT	Not serious	Serious	Serious	Not serious	Not likely	Moderate	The study was well designed, with proper randomisation, and all the outcomes mentioned were measured and reported. Slight concerns with the single randomisation process.

Abbreviations: GRADE, Grading of Recommendations Assessment, Development and Evaluation; RCT, randomised controlled trial.

**Figure 5:**
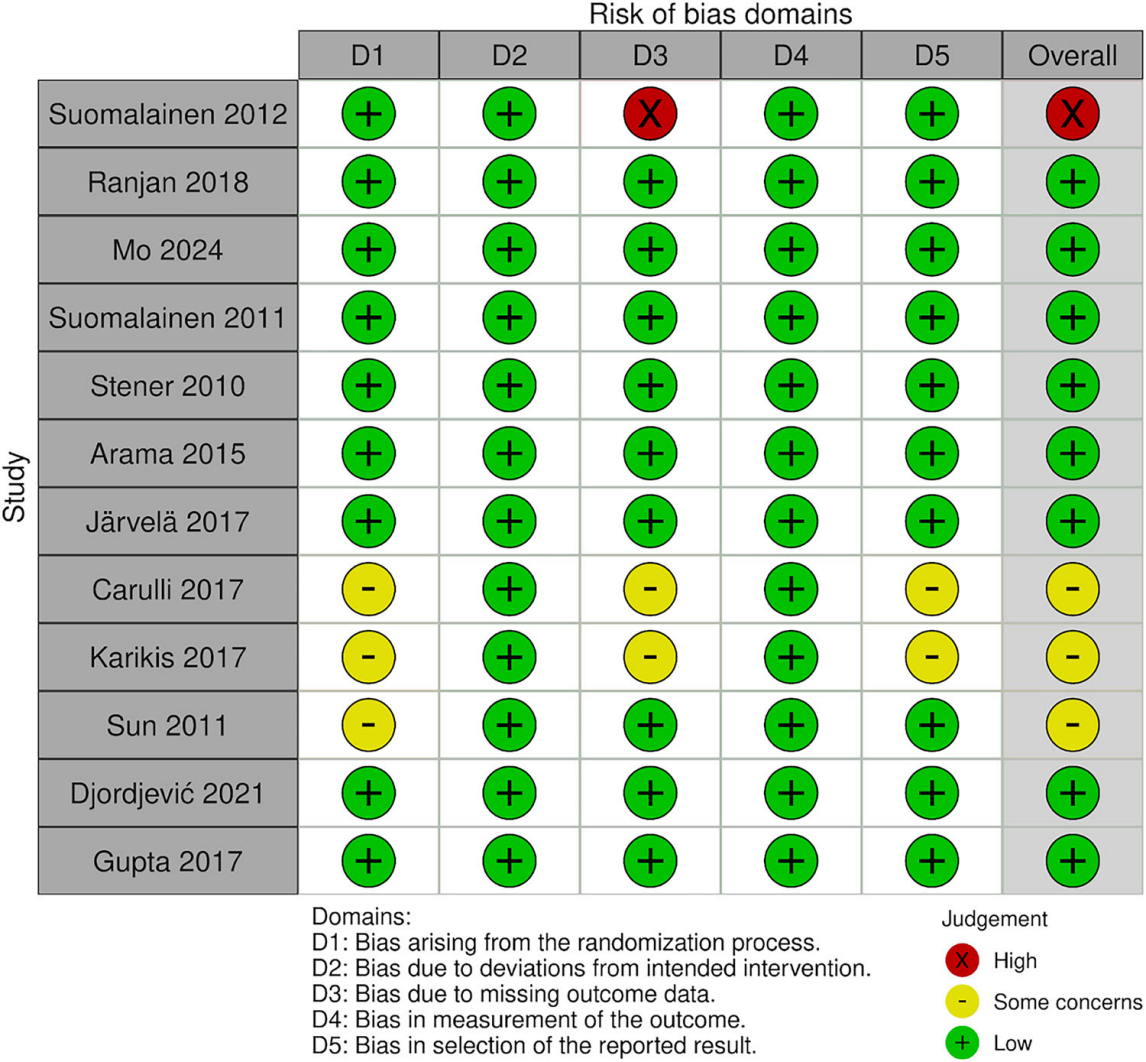
Traffic light plot of the RoB‐2 analysis of the included studies.

**Figure 6:**
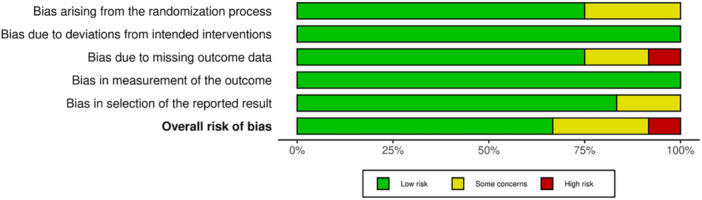
Summary plot of the RoB‐2 analysis of the included studies.

The same two authors performed a risk of bias analysis based on the Cochrane Risk of Bias Handbook [28]. RoB‐2 guidelines were followed for the 12 included randomised controlled trials for each domain of the RoB‐2 analysis. 8 studies were evaluated as low risk of bias for the methodology and reporting characteristics of the study. While three studies were rated with some concerns, and one study was considered high risk. Each domain that was evaluated for the included studies is represented in Figure 5, along with a summary plot as Figure 6 using the RobVis tool [18].

### Summary of findings

**Table 4:** Summary of findings regarding the 12 studies included in our review.

Title	Study ID (author, year)	Country	Study design	Recruitment period	Intervention	Number of patients	Age	Inclusion criteria	Outcomes collected	Main findings
Double‐bundle versus single‐bundle anterior cruciate ligament reconstruction	Suomalainen, Piia; 2012	Finland	RCT	2003–2005	Double bundle bioabsorbable ACL reconstruction vs. single bundle bioabsorbable ACL reconstruction vs. single bundle metallic ACL reconstruction	Double bundle: 20 Single bundle Bioabsorbable: 21 Single bundle Metallic: 24	Double Bundle: 34 ± 10 Single Bundle Bioabsorbable: 30 ± 8 Single Bundle Metallic: 33 ± 10	Primary ACL reconstruction with closed growth plates and lack of ligamentous injury to the contralateral knee	Pivot shift test with KT‐1000, IKDC, Lysholm Knee scores at the 5 year follow up	There are no statistically significant changes between the different surgery modalities in any of the three tests at the 5 year mark, but all surgeries showed significant improvement over pre‐surgery performance
In vivo comparison of a fixed loop (EndoButton CL) with an adjustable loop (TightRope RT) device for femoral fixation of the graft in ACL reconstruction: A prospective randomised study and a literature review	Ranjan, Rahul; 2018	India	RCT	November 2012–August 2014	Fixed loop endobutton vs. adjustable loop tightrope fixation devices	EndoButton: 52 Tightrope: 50	EndoButton: 28.7 ± 9.45 Tightrope: 30.4 ± 7.9	Clinical and radiological evidence of an ACL tear, ages 18–50, normal contralateral knee	IKCD, Lysholm, and ATT scores at 6 months and 2 years	Both procedures saw significant improvements from baseline; however they did not show significant differences in outcomes when comparing Endbutton to Tightrope
ACL reconstruction using quadrupled semitendinosus versus double‐stranded semitendinosus and gracilis autograft 2‐year results from a prospective randomised controlled study	Mo, Ingunn Fleten; 2024	Norway	RCT	April 2014–December 2019	All‐inside semitendinosus vs. traditional hamstring operation	All‐inside: 48 Traditional: 49	All‐inside: 34 ± 9.5 Traditional: 34 ± 10.1	Patient's age greater than 16, skeletal maturity, and no previous injury to same or contralateral knee	IKDC, KOOS, and TAS scores at baseline and 24 months	There are no significant differences in clinical outcomes between the 2 groups at the 2 year mark
Bioabsorbable versus titanium screws in anterior cruciate ligament reconstruction using hamstring autograft a prospective, blinded, randomised controlled trial with 5‐year follow‐up	Arama, Yuval; 2015	Australia	RCT	June 2002–October 2003	PLLA‐HA vs. Titanium	PLLA‐HA: 20 Titanium: 20	PLLA‐HA: 33 ± 7.2 Titanium: 29 ± 6.5	Patients with a primary anterior cruciate ligament reconstruction with 4‐stranded hamstring graft and written consent	IKCD, KT‐1000, single leg hop test at 2 and 5 years	There are no clinical differences in outcomes between the 2 categories at the 2 and 5 year marks
Double‐bundle versus single‐bundle anterior cruciate ligament reconstruction a prospective randomised study with 10‐year results	Jarvela, Sally; 2017	Finland	RCT	2003–2005	Double bundle bioabsorbable ACL reconstruction vs. single bundle bioabsorbable ACL reconstruction vs. single bundle metallic ACL reconstruction	Double bundle: 24 Single bundle Bioabsorbable: 23 Single bundle Metallic: 23	Double bundle: 34 ± 10 Single Bundle Bioabsorbable: 30 ± 8 Single Bundle Metallic: 33 ± 10	Primary ACL reconstruction with closed growth plates and lack of ligamentous injury to the contralateral knee	Pivot shift test with KT‐1000, IKDC, Lysholm Knee scores at the 10 year follow up	There are no statistically significant changes between the different surgery modalities in any of the three tests at the 10 year mark, but all surgeries showed significant improvement over pre‐surgery performance
Resorbable screw and sheath versus resorbable interference screw and staples for ACL reconstruction: a comparison of two tibial fixation methods	Carulli, Christian; 2016	Italy	RCT	2009 and 2012	BioIntrafix vs. BioRCI tibial screw and titanium staples	BioIntrafix: 45 BioRCI: 45	BioIntrafix: 31.0 (16–42) BioRCI: 31.8 (18–44)	Patients with unilateral isolated ACL ruptures who were candidates for an arthroscopically assisted reconstruction	KOOS, IKDC, and KT‐2000 scores at baseline, 6, 12, and 24 months	There are no significant clinical or radiological differences between BioIntrafix and BioRCI
Radiographic tibial tunnel assessment after anterior cruciate ligament reconstruction using hamstring tendon autografts and biocomposite screws: a prospective study with 5‐year follow‐up	Karikis, Ioannis; 2017	Sweden	RCT	March 2008–September 2009	Single bundle	Single bundle: 51	Single bundle: 28 ± 8.5	Patients 18 years or older with a unilateral ACL injury	KT‐1000, pivot‐shift test, ROM, Lysholm knee scoring scale, and Tegner activity preoperatively and 5 years later	This study group showed improved clinical outcomes compared to their preoperative results
Arthroscopic reconstruction of the anterior cruciate ligament with hamstring tendon autograft and fresh‐frozen allograft: A prospective, randomised controlled study	Sun, Kang; 2011	China	RCT	2000–2004	Hamstring tendon autograft vs. hamstring tendon allograft	Autograft: 91 Allograft: 95	Autograft: 29.6 ± 6.9 Allograft: 31.2 ± 8.3	Patients with no previous injury of surgery to the affected knee, no multiple ligamentous injuries, no malalignment, and ability to complete the study protocol	Lachman test, Anterior Drawer test, pivot shift test, varus and valgus stress test, KT‐2000, IKDC, Daniel 1‐legged hop test, Harner vertical jump test, ROM testing with an average follow up time of 7.8 years	There are no significant differences in the clinical outcomes between autograft and allograft patients, except for Allografts having a shorter operation time
Outcome of hamstring autograft with preserved insertions compared with free hamstring autograft in anterior cruciate ligament surgery at 2‐year follow‐up	Gupta, Ravi; 2017	India	RCT	April 2012–April 2014	Hamstring tendon autograft with preserved insertions vs. free hamstring tendon autograft	Insertions: 55 Free Hamstring: 55	Insertions: 27.0 ± 7.5 Free Hamstring: 27.2 ± 5.7	Patients who are adult professional athletes with an ACL injury greater than 3 weeks old without any signs of knee inflation	Lachman test, Anterior Drawer test, Pivot Shift test, KT‐1000 test, Cinncinati knee score, Tegner activities score were assessed preoperatively, 3 months, 6 months, 12 months, and 24 months minimum	Hamstring autografts with preserved insertions had statistically better superior anterior stability, improved functional outcomes, and a closer return to pre‐injury sporting activity, however, no clinically significant difference was proven
Double‐bundle versus single‐bundle anterior cruciate ligament reconstruction: randomised clinical and magnetic resonance imaging study with 2‐year follow‐up	Suomalainen, Pia; 2011	Finland	RCT	March 2003– February 2008	Single‐bundle technique with aperture interference screw fixation vs. double‐bundle technique with aperture interference screw fixation	Single Bundle: 60 Double Bundle: 61	Single Bundle: 32 ± 10 Double Bundle: 32 ± 10	Patients with a primary ACL reconstruction, closed growth plates, and absence of ligament injury to the opposite knee	Clinical and MRI evaluation, KT‐1000, IKCD, and Lysholm knee score	The revision rate was significantly lower in patients who received double bundle technique than those who received single bundle
Application of two types of suspensory fixation in reconstruction of anterior cruciate ligament with a semitendinosus‐gracilis graft – A randomised prospective study	Djordjević, Dušan; 2021	Serbia	RCT	January 2015–December 2017	Femoral tunnel graft fixation with an adjustable length loop implant vs. Fixed length loop implant	Adjustable: 30 Fixed: 30	Adjustable: 26.87 ± 6.388 Fixed: 27.87 ± 6.902	Patients with a unilateral lesion of the ACL less than 10 months prior to reconstruction, with or without a minor lesion of the medial or lateral meniscus, without arthritic changes and neuromuscular diseases; with 1 A femoral tunnel position, with 4B tibial tunnel position; with a willingness to participate in the study and to adhere to the rules for clinical and functional evaluation and for rehabilitation	KT‐1000, Lysholm score, IKDC‐2000, Lachman test, Pivot‐shift test	The functional results of both the fixed and adjustable implants were the same
A long‐term, prospective, randomised study comparing biodegradable and metal interference screws in anterior cruciate ligament reconstruction surgery: radiographic results and clinical outcome	Stener, Sven; 2010	Sweden	RCT	January 1999–March 2000	Poly‐l‐lactide acid (PLLA) Screw Fixation vs. Metal Interference Screw fixation	PLLA: 33 Metal: 31	PLLA: 26 (16‐38) Metal: 27 (16‐46)	Unilateral ACL rupture verified clinically by a positive Lachman test and a positive pivot‐shift test or through a previous diagnostic arthroscopy	KT‐1000, Lysholm score, Tenger level, single‐leg hop test	There were significantly larger bone tunnels on the femoral side with the PLLA group compared to the metal group

*Note*: Ages have been expressed as mean ± SD, or mean (range).

Abbreviations: ATT, anterior tibial translation; IKDC, International Knee Documentation Committee; KOOS, Knee Injury and Osteoarthritis Outcome score; KT‐1000, medical device that accesses knee stability; PLLA‐HA, poly‐l‐lactic acid‐hyaluronic acid; RCT, randomised controlled trial; TAS, Tegner Activity Scale.

## DISCUSSION

The results of this review found that the utilisation of CB fixation of autograft HT ACLR on the femoral aspect yielded greater reduction in KT‐1000 side‐to‐side differences at 2‐years postoperatively. Additionally, significant improvements in Lysholm and Tegner scores at 5–10 year follow‐up were found in the CB cohort compared to the IS group. HT autografts are a commonly utilised graft that has fallen in and out of favour over the past 30 years [8, 15]. Despite their increased use, there is conflicting evidence about the optimal form of femoral fixation for these grafts.

In 2021, a systematic review and meta‐analysis was conducted by Yan et al, comparing three femoral fixation methods for HT autografts: IS, CB and cortical bone pins [35]. Their results found no statistically significant differences in outcomes between CB and IS. This discrepancy in the findings may be due to several factors. The main factor being that the previously published review only included two head‐to‐head studies to compare the two fixation methods, yielding a smaller, and possibly less generalisable sample size. Additionally, for these two fixation methods, the review had a follow‐up of approximately 2–3 years, and compared IKDC scores, rather than KT‐1000 side‐to‐side differences, Lysholm, or Tegner scores. Outside of the small sample size making it difficult to extrapolate the data more broadly, one of the two studies included was non‐randomised, further complicating the validity of the data. As such, the present meta‐analysis pooled data from ten studies, which has resulted in a greater sample size.

One possible explanation of our results pertains to the circumferential healing potential of the grafts within the femoral tunnel with CB suspensory fixation. CB fixation provides significantly higher yield load and ultimate failure load than IS [33]. The higher yield load observed in CB may result in less deformation in the structural integrity of the ACLR, potentially allowing for the fixation to work as intended for a longer period of time. Similarly, the higher ultimate failure load may reduce the need for ACLR revisions, which are known to reduce functional outcomes and patient satisfaction, while increasing the risk of osteoarthritis of the knee [12]. On the contrary, IS fixation compresses both the device and the graft, leading to possible deformation of the fixation device, while causing biomechanical compromise of the fixated grafts, as observed in an in vitro study conducted by Teng et al. [32].

Comparing CB to IS femoral fixation in ACLR with all soft‐tissue HT autografts, CB provided greater improvements in knee stability at 2‐years, and superior knee function with increased levels of activity at 5–10 years postoperation. However, due to the significant amount of heterogeneity observed in our analysis, surgeons must use their own discretion regarding which femoral fixation method is most appropriate for each individual patient. The results of our review promote the necessity of further, large‐scale RCTs, directly comparing CB to IS while assessing for the following outcomes: KT‐1000 side‐to‐side differences, International Knee Documentation (IKDC) scores, Lysholm scores, Tegner scores, VAS scores, pivot shift tests, retear rates and revision rates.

### Limitations

According to the RoB‐2 analysis, one included study was of high concern for bias, three studies were of moderate concern, and eight studies were of low concern, with bias arising from randomisation processes, missing outcome data, and the available reported results. The GRADE analysis conducted on each of the included studies portrayed three studies to be of low quality of evidence, with the remaining nine studies yielding moderate qualities of evidence.

Regarding the design of our review, there are some limitations. Not all of the RCTs we included and analysed were directly comparing CB to IS. If an RCT was comparing one of the interventions of interest to another intervention that was not of interest, we collected and analysed the data only regarding our fixation method of interest. Additionally, our review analyzes different outcomes at different timelines, with KT‐1000 side‐to‐side differences being assessed at 2‐year follow‐up, while Lysholm and Tegner scores were assessed at 5–10 year follow‐up. Generalisability to other age groups may also pose a limitation to our review, as the mean ages at 2‐year and 5–10 year follow‐ups were 30.5 ± 8.7 and 30.2 ± 8.1, respectively. Moreover, our review did not compare revision and complicate rates for each intervention, largely due to unavailability of data in each included study. Importantly, this review focused exclusively on femoral fixation methods and did not compare tibial fixation techniques, which may also influence overall ACLR outcomes. Furthermore, we pooled data regarding fixed and adjustable loops (CB group) as well as biocomposite and metallic screws (IS group), which may have resulted in confounding by fixation type. However, previously published reviews have shown no significant differences between the mentioned subgroups [9, 34].

Lastly, the degree of heterogeneity of our review is significant in regards to the outcomes KT‐1000 (*I* ^2^ = 0.99) Lysholm scores (*I* ^2^ = 0.76) and Tegner scores (*I* ^2^ = 0.97). This may be due to our review including RCTs that were not directly head‐to‐head comparisons between CB and IS. Additionally, included studies may have differed in regards to surgeon experience, and post‐operation rehabilitation techniques.

## CONCLUSION

Compared to IS fixation, CB fixation of HT autografts on the femoral aspect of ACLR may yield more symmetric knee laxity at 2‐years postoperatively, and greater functionality and activity at the 5–10 year postoperative follow‐up.

## AUTHOR CONTRIBUTIONS

Jonathan Elias conceived of the presented idea. Jonathan Elias, Mitchell Kaplan, and MB selected studies. Mitchell Kaplan, Michael Bickford, Thomas Oliver, and Kunal Shah extracted data from the studies included. Jonathan Elias completed the statistics. Kunal Shah and Thomas Oliver completed the GRADE and bias assessment for each included study. Jonathan Elias, Mitchell Kaplan, Michael Bickford, Thomas Oliver, Kunal Shah, Elizabeth Ford, and Sean McMillan participated in the write‐up of the manuscript. Elizabeth Ford and Sean McMillan provided areas for improvement of the study concept and provided their input in their respective areas of expertise. All authors have reviewed and endorsed the final manuscript.

## CONFLICT OF INTEREST STATEMENT

The authors declare no conflicts of interest.

## ETHICS STATEMENT

The review was not registered with an institutional review board, as there was no contact with human subjects or access to personal information.

## Data Availability

The data extracted from each study is publicly available at each study's respective journal.
